# Impact of Preoperative Psychiatric Profile in Bariatric Surgery on Long-term Weight Outcome

**DOI:** 10.1007/s11695-023-06595-2

**Published:** 2023-05-05

**Authors:** Anouk Lüscher, Nathalie Vionnet, Michael Amiguet, Dionysios Chartoumpekis, Styliani Mantziari, Johanna Frantz, Lucie Favre

**Affiliations:** 1 Faculty of Biology and Medicine, University of Lausanne, Lausanne, Switzerland; 2 Service of Endocrinology, Diabetes and Metabolism, Lausanne University Hospital, Lausanne, Switzerland; 3 Center for Primary Care and Public Health (Unisanté), University of Lausanne, Lausanne, Switzerland; 4 Service of Visceral Surgery, Lausanne University Hospital, Lausanne, Switzerland; 5 Liaison Psychiatry, Lausanne University Hospital, Lausanne, Switzerland; 6 Present Address: Centre Hospitalier Universitaire Vaudois, CHUV, Division of Endocrinology, Diabetology and Metabolism, Lausanne University Hospital, Rue St Martin 3, CH-1003 Lausanne, Switzerland

**Keywords:** Bariatric surgery, Roux-en-Y gastric bypass, Anxiety, Weight loss, Outcomes

## Abstract

**Background:**

Conflicting results have been reported regarding the predictive value of preoperative psychological assessment and weight outcome after bariatric surgery. This might be attributed to different factors affecting early weight loss and long-term weight loss. Herein, we investigated whether preoperative psychiatric profile was associated with preoperative BMI and with both early (1 year) and long-term (5 years) weight loss after Roux-en-Y gastric bypass (RYGB).

**Methods:**

Prospective observational cohort study of patients undergoing RYGB between 2013 and 2019. Symptoms related to anxiety, depression, eating disorder, and alcohol use disorders were assessed by employing validated, specific psychometric tests (STAI-S/T, BDI-II, BITE, AUDIT-C) prior to surgery. Pre-operative BMI, early weight loss (1 year), and long-term weight evolution (up to 5 years) were registered.

**Results:**

Two hundred thirty six patients (81% women) were included in the present study. Linear longitudinal mixed model showed a significant effect of preoperative high anxiety (STAI-S) on long-term weight outcome, after controlling for gender, age and type 2 diabetes. Patient with high preoperative anxiety score regained weight faster than those experiencing low anxiety (each year percent excess BMI loss (%EBMIL) − 4.02%, ± 1.72, *p* = 0.021). No other pre-operative psychiatric symptoms have been shown to have an impact on long-term weight loss. In addition, no significant association was found between any of the pre-operative psychiatric variables and pre-operative BMI, or early weight loss (%EBMIL) at 1-year post-RYGB.

**Conclusion:**

Herein we identified high anxiety score (STAI-S) as a predictor for long-term weight regain. Thus, long-term psychiatric surveillance of these patients and the development of tailored management tools could serve as a means to prevent weight regain.

**Graphical abstract:**

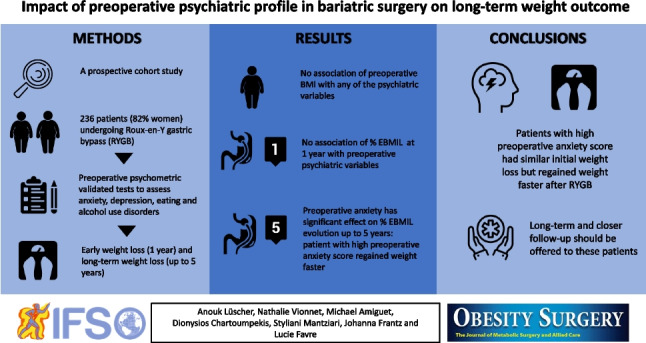

## Introduction

Obesity is a major public health problem [1], and bariatric surgery has been proven to be the most effective treatment option currently available for severe obesity (BMI > 35 kg/m^2^) [2]. However, metabolic and bariatric surgery [3] does not guarantee sufficient weight loss or long-term weight stability, as 20 to 30% of patients experience insufficient weight loss or significant weight regain [4, 5]. Optimizing weight loss remains a challenge in the post-operative management of bariatric patients. Predictors that may influence post-operative weight loss include demographic, genetic, behavioral, socio-economic, and biological factors but to date, contradicting evidence regarding these associations has been reported [4, 6-9].

Mental health problems are common among patients suffering from obesity that seek bariatric surgery [10, 11]. Anxiety, depression, and binge eating disorders are the most prevalent psychiatric disorders reported in 12%, 19%, and 17% of bariatric surgery candidates, respectively [12]. Studies aiming to identify a predictive relationship between preoperative mental health symptoms and weight loss after surgery have yielded conflicting results [13-19] as have systematic reviews addressing this topic [8, 12]. These discrepancies may be explained by the fact that the preoperative psychiatric factors that influence initial weight loss may differ from those influencing weight regain. In fact, the majority of studies on this topic have assessed preoperative psychiatric conditions in relation to short-term weight change, up to 1 year—after surgery [13, 16, 19-21], and only a few have analyzed possible associations with long-term weight outcome, from 3 years post-surgery onwards [14, 15, 22].

In the present study, we hypothesized that preoperative mental health symptoms were associated with preoperative BMI and might differently influence initial weight loss and long-term weight loss, a combination of initial weight loss and subsequent weight regain. We therefore examined the association of mental health symptoms with preoperative BMI, we evaluated the association of a set of preoperative conditions on early (1 year) and long-term (up to 5 years) weight loss after Roux-en-Y gastric bypass (RYGB), and we explored the utility of a preoperative psychiatric profile to predict weight trajectories post-RYGB.

## Methods

### Study Population and Design

This is a prospective, observational cohort study in a tertiary referral University hospital (Lausanne University Hospital). The cohort included 310 patients aged ≥ 18 who underwent a laparoscopic Roux-en-Y gastric bypass (RYGB) as primary bariatric procedure between June 2013 and August 2019. From the initial sample size, 74 patients were excluded for not properly completing the self-report questionnaires included in the psychiatric evaluation, most often because of a language barrier. Demographics and clinical characteristics were similar for gender, weight, BMI, and comorbidities between included and excluded participants. Only age was significantly different, with excluded patients being older (44.3 versus 40.8, *p* 0.01).

Prior to surgical intervention, patients were evaluated and prepared by a multidisciplinary team and met the eligibility criteria for bariatric surgery as defined by the Swiss Society for the Study of Morbid Obesity and Metabolic Disorders (SMOB) [23].

### Surgical Intervention

The surgical technique is standardized in our center, and all patients had laparoscopic RYGB with a gastric pouch (< 25 mL), an alimentary limb of 100 cm and a biliopancreatic limb of 50 cm.

### Measures

Psychiatric assessment included measures of symptoms related to depression, anxiety, eating disorders, and alcohol consumption through the following validated, self-reporting psychometric tests.

#### **Beck Depression Inventory II the 21-Item BDI-II** [24]

This 21-item BDI-II assesses the presence and the severity of depression. Grades of depression are based on score cutoffs [25]: minimal (0–13 points), mild (14–19 points), moderate-severe (20–28 points), and severe (28–63 points). Subjects with BDI-II raw score ≥ 14 were considered to have symptoms suggestive of clinical depression [24].

#### **Spielberger State-Trait Anxiety Inventory** [26] 

This test consists of two separate sub-scales that measure trait anxiety (STAI-T), a general propensity to be anxious in different situations, and state anxiety (STAI-S), the degree of anxiety felt at the current situation. Each subscale uses a 4-point Likert scale for its 20 items. Scores 20–39 indicate low anxiety, 40–59 moderate anxiety, and 60–80 high anxiety.

#### **Bulimic Investigatory Test, Edinburgh** [27]

This 33-item scale is a validated tool for the detection of binge eating and bulimia nervosa consisting of two subscales. The symptom scale (30 items) measures the degree of symptoms with scores ranging from 0 to 30. Score < 10 indicates normal eating behavior, 10 to 19 abnormal eating behavior, ≥ 20 highly disordered eating pattern and presence of binge eating. The severity scale (3 items) provides an index of the severity of the eating disorder with score ranging from 0 to 30. Score < 5 indicates absence of bulimic symptoms, ≥ 5 presence of clinically relevant bulimic symptoms, ≥ 10 high degree severity.

#### **Alcohol Use Disorders Identification Test-Consumption** [28] 

This brief 3-item screening test is widely used for detecting risky alcohol use. The scores range from zero to 12 points. It is positive in men with a score of ≥ 4, in women with a score ≥ 3.

### Postoperative Outcome

The amount of weight loss after bariatric surgery was expressed in terms of percentage of total weight loss (%TWL) and percentage of excess BMI loss (%EBMIL). Weight was measured preoperatively and yearly up to 5 years after surgery by our team during in-person visits while patients were dressed in light clothing. The %EBMIL was used as main outcome measure and calculated with the formula %EBMIL = ([pre-operative BMI-current BMI]/[pre-operative BMI-ideal BMI]) × 100; a BMI of 25 kg/m^2^ was considers as the ideal BMI.

### Statistical Analyses

Descriptive analyses were performed for each variable. A multiple regression analysis of pre-operative BMI was conducted in order to assess its association with the pre-operative psychiatric variables. On top of psychiatric explanatory variables, the following adjustment variables were included gender, age in four categories based on quartiles ([21, 33], (33,40], (40, 49], (49, 68]), and type 2 diabetes. In this model, the outcome was log-transformed prior to modeling, in order to better meet the hypothesis of normality in errors. A linear longitudinal mixed model was built in order to assess the impact of the pre-operative psychiatric variables on post-operative BMI evolution. Psychiatric explanatory variables and adjustment variables were the same as in the pre-operative BMI model, but the outcome for this analysis was %EBMIL. From the longitudinal model, we deduced the most and least favorable psychiatric profiles in terms of %EBMIL at 1 year and in terms %EBMIL up to 5 years (slope of %EBMIL evolution with time), thus identifying four profiles. The profiles were deduced from the longitudinal model coefficients, by identifying the covariate patterns producing the best and the worst predictions in terms of % EBMIL at 1 year and of subsequent evolution up to 5 years. As most of the coefficients are not statistically significant, the patterns reflect uncertain information and should not be interpreted in detail. Their main purpose is to assess the predictive power of the pre-operative psychiatric variables, by identifying the most contrasted predictions that can be obtained. In order to quantify the differences between the profiles, prediction bands were generated.

Finally, to further investigate the predictive power of the psychiatric profile, we evaluated the width reduction of the prediction bands (i.e., the gain in precision of prediction) offered by the psychiatric variables, by comparing our model to a model without these variables. At each time point, we considered the ratio of the widths of the prediction bands calculated from each of the two models, and built a confidence interval for this ratio via a resampling method (bootstrap). The bootstrap was implemented with 1000 resamplings with replacement. Statistical analyses were conducted using R statistical software version 3.4.2 (R Core Team 2017) [29].

## Results

**Fig. 1:**
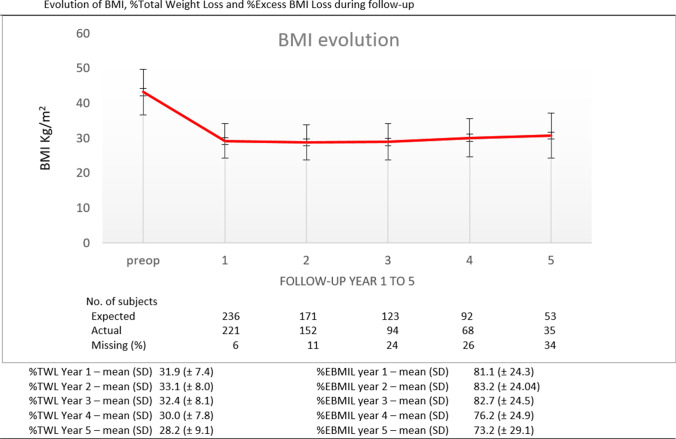
Preoperative BMI and evolution of BMI, percent of total weight loss (%TWL) and percent excess BMI loss (%EBMI) during follow-up. Expected: no of patients who actually reached the different analysis time. Actual: no of patient among those who reached the analysis time with anthropometric data available. Missing %: percentage of patients with missing values at each time point

A total of 236 patients were included in the study. Pre-operative variables and results of the psychometric evaluation are shown in Table 1. Preoperative psychometric tests showed that 18% patients had symptoms of depression, 48% had anxiety, 27% a general propensity to be anxious, 19% had an abnormal eating behavior, and 17% presented risky alcohol drinking habits. Evolution of the BMI during follow-up and missing weight data can be found in Fig. 1. This is a longitudinal cohort study and the duration of the follow-up differs among participants. Data at 1 year were available for 221 patients out of 236 (missing 6%) and at 5 years for 35 participants out of 54 (missing 34%) (Fig. 1).

**Table 1:** Baseline characteristics of patients

Demographic and clinical characteristics	Total (*n* = 236)	Missing (%)
Age (year) – mean (SD)	40.8 (± 10.1)	-
Gender- *n* (%)		
Female	192 (81.4)	-
Male	44 (18.6)	-
Weight (kg) – mean (SD)	118.8 (± 23.8)	-
BMI (kg/m^2^) – mean (SD)	43.2 (± 6.5)	-
Comorbidities – *n* (%)		
Diabetes mellitus	48 (20.4)	-
Arterial hypertension	90 (38.1)	-
Dyslipidemia	126 (53.4)	-
Coronary artery disease	3 (1.3)	-
Hyperuricemia	39 (16.5)	-
Hypothyroidism	25 (10.6)	-
Psychiatric variables		Missing (%)
BITE probability – *n* (%)		0.4
Normal	190 (80.9)	
Unusual eating pattern	35 (14.9)	
Binge eating	10 (4.3)	
BITE severity – *n* (%)		0.4
Normal	212 (90.2)	
Clinically significant	23 (9.8)	
BDI-II depression – *n* (%)		1.2
Minimal	191 (82)	
Mild	20 (8.6)	
Moderate	15 (6.4)	
Severe	7 (3)	
STAI anxiety state – *n* (%)		0.4
Low anxiety	123 (52.3)	
Moderate anxiety	77 (32.7)	
High anxiety	35 (15)	
STAI anxiety trait – *n* (%)		0.8
Low anxiety	170 (72.6)	
Moderate anxiety	34 (14.5)	
High anxiety	30 (12.8)	
AUDIT-C – *n* (%)		3
Negative	192 (83)	

*BMI* body mass index [kg/m^2^], *BITE* Bulimic Investigatory Test Edinburgh, *BDI-II* Beck Depression Inventory II, *STAI* Spielberger State-Trait Anxiety Inventory, *AUDIT-C* Alcohol Use Disorders Identification Test-Consumption

### Preoperative Mental Health Symptoms and Preoperative BMI

Multiple regression analysis showed no significant association of preoperative BMI with any of the psychiatric variables (Table 2). The youngest patients (21 to 33 years) had a significantly higher pre-operative BMI than patients of older age groups.

**Table 2:** Multiple regression analysis of log (pre-operative) BMI

Exp (beta)	Inferior limit	Superior limit	*p* value	
BITE ED probability: unusual eating pattern	1.00	0.95	1.05	0.959
BITE ED probability: binge eating	0.99	0.87	1.13	0.913
BITE ED severity: clinically significant	1.02	0.95	1.09	0.605
*Reference category: normal				
BDI depression: mild	1.01	0.93	1.09	0.809
BDI depression: moderate	0.99	0.90	1.09	0.835
BDI depression: severe	0.94	0.82	1.07	0.317
*Reference category: minimal				
STAI anxiety state: moderate anxiety	1.01	0.96	1.05	0.763
STAI anxiety state: high anxiety	0.97	0.90	1.05	0.468
*Reference category: low anxiety				
STAI anxiety trait: moderate anxiety	1.01	0.95	1.08	0.718
STAI anxiety trait: high anxiety	1.06	0.97	1.15	0.221
*Reference category: low anxiety				
AUDIT-C positive	1.01	0.96	1.06	0.742
*Reference category: negative				
Gender M	1.03	0.98	1.08	0.232
*Reference category: F				
BS age category [33–40]	0.95	0.90	1.00	0.049
BS age category [40—49]	0.93	0.88	0.98	0.006
BS age category [49—70]	0.94	0.89	0.99	0.028
*Reference category: 21–33 y.o				
Type 2 Diabetes	1.02	0.98	1.08	0.334
*Reference category: absence				

*BITE* Bulimic Investigatory Test Edinburgh, *BDI-II* Beck Depression Inventory II, *STAI* Spielberger State-Trait Anxiety Inventory, *AUDIT-C* Alcohol Use Disorders Identification Test-Consumption

### Preoperative Mental Health Symptoms and Bariatric Outcomes

The linear longitudinal mixed model showed no significant association of %EBMIL at 1 year with any of the included psychiatric variables (Table 3). In terms of %EBMIL evolution to year 5, anxiety state has a significant effect as those with high anxiety score regain weight faster; each year, 4.02% [CI = (0.65–7.39] is regained on top of what is regained by patients with low anxiety score.

**Table 3:** Linear Longitudinal Mixed Model of EBMIL evolution

EBMIL at year 1	Estimate	Standard Error	*p*-value
BITE ED probability: unusual eating pattern	3.06	4.75	0.520
BITE ED probability: binge eating	-13.41	11.35	0.239
BITE ED severity: clinically significant	-6.14	6.11	0.317
*Reference category: normal			
BDI depression: mild	-1.99	6.56	0.762
BDI depression: moderate-severe	5.57	7.80	0.476
*Reference category: minimal			
STAI anxiety state: moderate anxiety	2.63	3.68	0.476
STAI anxiety state: high anxiety	3.82	6.47	0.555
*Reference category: low anxiety			
STAI anxiety trait: moderate anxiety	-5.26	5.23	0.316
STAI anxiety trait: high anxiety	-8.06	7.36	0.275
*Reference category: low anxiety			
AUDIT-C positive	1.45	4.39	0.743
*Reference category: negative			
Gender M	-15.29	4.33	0.001
*Reference category: F			
BS age category [33–40]	8.72	4.45	0.051
BS age category [40—49]	10.39	4.71	0.029
BS age category [49—70]	5.64	4.82	0.243
*Reference category: 21–33 y.o			
Type 2 Diabetes	-3.97	4.21	0.346
*Reference category: absence			
EBMIL evolution until year 5	Estimate	Standard Error	*p*-value
BITE ED probability: unusual eating pattern	-2.40	1.23	0.055
BITE ED probability: binge eating	-4.73	2.73	0.085
BITE ED severity: clinically significant	2.90	1.60	0.073
BDI depression: mild	0.27	1.78	0.879
BDI depression: moderate-severe	-0.31	2.61	0.907
STAI anxiety state: moderate anxiety	1.33	1.01	0.188
STAI anxiety state: high anxiety	-4.02	1.72	0.021
STAI anxiety trait: moderate anxiety	0.27	1.34	0.844
STAI anxiety trait: high anxiety	1.67	2.25	0.460
AUDIT-C: positive	-0.81	1.11	0.471

*BITE* Bulimic Investigatory Test Edinburgh, *BDI-II* Beck Depression Inventory II, *STAI* Spielberger State-Trait Anxiety Inventory, *AUDIT-C* Alcohol Use Disorders Identification Test-Consumption

Regarding eating habits, a trend is observed with yearly weight regain in the presence of unusual eating habits (2.4%, CI = [− 0.02–4.81] in addition to what is regained by patients with normal eating habits), without reaching statistical significance (*p* = 0.055). Among adjustment variables, gender showed significant impact on %EBMIL at 1 year, with lower weight loss in men by 15.29% (CI = [23.78–6.8]). Furthermore, we found the age group [40–49] to be significantly associated with more favorable %EBMIL at 1 year, patients in this age group lost 10.39% (CI = [1.16–19.63]) more during the first year compared to the 21–33 year old.

### Preoperative Psychiatric Profile to Predict Weight Outcome

**Fig. 2:**
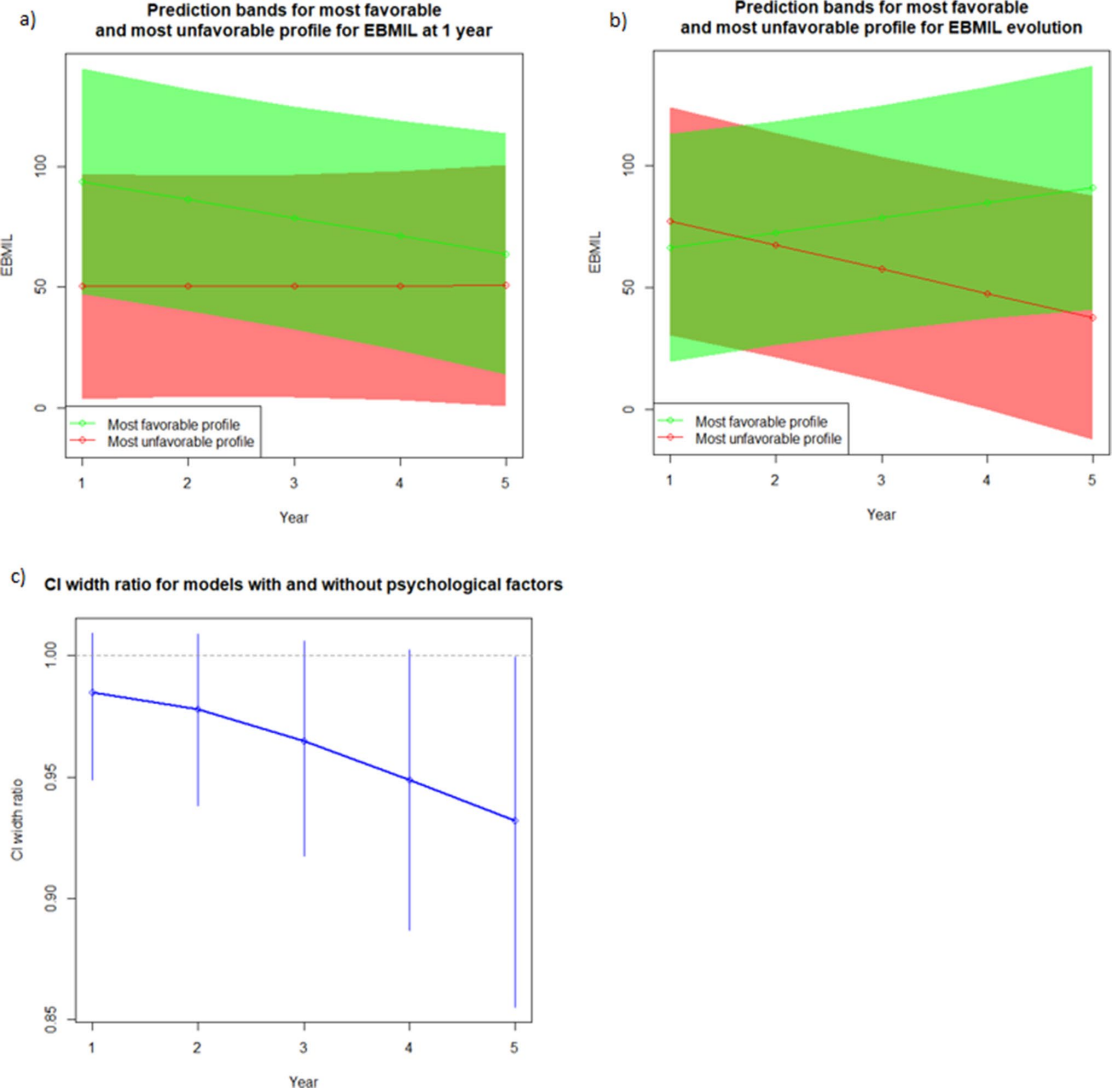
Prediction bands for most favorable and most unfavorable psychiatric profile for EBMIL **a** for EBMIL at 1 year, **b** for EBMI evolution, **c** CI width ratio for models with and without psychological factors

We established the most and least favorable psychiatric profiles in regards to short- and long-term weight loss and sought to assess their ability to predict weight loss. The difference in %EBMIL at 1 year, as well as the yearly evolution of %EBMIL, between the corresponding profiles was statistically significant (43.5%, CI = (4.99–82.01) and 16.5%, CI = (2.93–29.16) respectively (Table 4). The important overlapping of the prediction bands for these profiles (Fig. 2a and b), illustrates however the poor predictive value of the psychiatric variables. The resampling method (Fig. 2c) shows that the mean gain in precision offered by the available psychiatric variables is low over the considered period (mean ratio of widths of prediction bands ranges from 0.94 to 0.98).

**Table 4:** Preoperative psychiatric profile to predict weight outcome

Most and least favorable psychiatric profile in terms of EBMIL at 1 year		
	Least Favorable Profile	Most Favorable Profile
BITE ED probability	clinically significant	normal
BITE ED severity	binge eating	unusual eating pattern
BDI depression	mild	moderate-severe
STAI anxiety state	low anxiety	high anxiety
STAI anxiety trait	high anxiety	low anxiety
AUDIT-C	negative	positive
Mean EBMIL	50.23	93.73
*EBMIL = *excess BMI loss [%]*		
Difference	43.50	
CI Difference	4.99 – 82.01	
*P*-value Difference	0.03	
Most and least favorable psychiatric profile in terms of EBMIL evolution		
	Least Favorable Profile	Most Favorable Profile
BITE ED probability	normal	clinically significant
BITE ED severity	binge eating	normal
BDI depression	moderate-severe	mild
STAI anxiety state	high anxiety	moderate anxiety
STAI anxiety trait	low anxiety	high anxiety
AUDIT-C	positive	negative
Mean Slope	-11.68	4.37
Difference	16.05	
CI Difference	2.93 – 29.16	
*P*-value Difference	0.02	

*BITE* Bulimic Investigatory Test Edinburgh, *BDI-II* Beck Depression Inventory II, *STAI* Spielberger State-Trait Anxiety Inventory, *AUDIT-C* Alcohol Use Disorders Identification Test-Consumption

## Discussion

The first aim of our study was to assess the relationship between preoperative psychiatric profile and preoperative BMI. Overall, the prevalence of depressive, anxiety and eating disorders symptoms in our cohort was high, in accordance with previously published findings [10, 11, 30-33]. Yet, our data did not support a relationship of these symptoms with the preoperative BMI. In our cohort, 17% of patients presented risky alcohol drinking habits before surgery, a finding similar to those found in other studies [34]. It should be noted that, according to the usual recommendations, patients with alcohol dependence are not represented in this study, as bariatric surgery is contraindicated in cases of proven dependence on alcohol or other substances.

Secondly, we investigated the role of pre-operative psychiatric symptoms on short- and long-term weight outcome. We found that high state anxiety had no impact on initial weight loss but was associated with an increased risk of long-term weight regain. Studies examining associations between weight change after bariatric surgery and anxiety are scarce [15, 35, 36]. Legenbauer et al. [35] and de Zwaan et al. [15] found that patients with anxiety disorders at baseline had a less favorable weight trajectory, but in both studies, the surgical procedures performed were solely or mainly adjustable gastric banding (100% and 81% respectively). Recently, Aylward et al. [36] reported similar results in patients with RYGB or SG, demonstrating that higher anxiety was associated with less weight loss over time (30 months follow-up). In accordance with this study, we have demonstrated a negative impact of state anxiety on long-term weight results. There are several potential explanations for the link between anxiety and weight outcome after bariatric surgery. The level of physical activity that is decreased in patients with anxiety [37] may be a factor involved, as this is a major determinant of weight loss maintenance after bariatric surgery [38]. Patients with anxiety may be more susceptible to adopt dysfunctional eating behaviors as eating might be used as a coping mechanism to deal with negative effects caused by a specific stressful situation. These hypotheses are supported by scarce clinical data to this day and need to be confirmed by further clinical research. Our results highlight the influence of anxiety on long-term weight outcome and therefore the need to assess this disorder prior to bariatric surgery and to maintain closer follow-up of these patients.

Our study did not show any significant association between pre-surgery depressive symptoms or risky alcohol use on short-term and long-term weight outcomes. This is in accordance with previous studies reporting that depressive symptoms, assessed either through psychometric tests [4, 33, 39, 40] or structured clinical interview [13, 15], did not predict short and/or long-term weight loss (up to 3 years). Similarly, preoperative risky alcohol use does not seem to affect the weight outcomes in the short [13] and long term [4].

In our study, eating behavior did not seem to have a significant impact on short-term weight change. A trend for unusual eating pattern to impact long-term weight loss was observed. The absence of influence of preoperative eating behavior on initial weight loss is in agreement with some previous reports [16, 41, 42], and its potential impact on long-term weight regain is in accordance with a few others [43, 44]. However, all these previous studies had enrolled patients who underwent purely restrictive procedures (vertical banded gastroplasty, adjustable gastric banding) [42-44] or a mix of restrictive procedure and RYGB [4, 16, 41]. This is an important limitation when considering the dramatic changes in eating patterns inherent to the different bariatric procedures.

In our multivariable model, the gender was found to have an impact on EBMIL at 1 year post-surgery (*p* < 0.05), with lower EBMIL in men. The effects of gender in weight loss after bariatric surgery have been a matter of debate [8, 45-47].

In an attempt to assess the predictive power of the pre-operative psychiatric symptoms on post-surgery weight outcomes, we devised profiles with the most contrasted predictions regardless of the covariate patterns. We thus identified profiles that were significantly associated with short- or long-term weight loss. However, these profiles had a poor predictive value as shown by the important overlapping of the associated prediction bands.

### Strengths and Limitations

The strengths of this study are the analysis of a specific population who underwent a single type of bariatric surgery, the use of validated psychometric tests and the longitudinal perspective with a long-term follow-up. We have controlled our analysis for other variables that may affect weight trajectory. Its main limitation is that data are currently being collected and at the time the analyses presented here were carried out, the available information provided limited power to detect effects smaller than 5% yearly regain (in units of %EBMIL) during the 5-year follow up period (the half widths of CI’s range from 1.9 to 5.1% for these effects). Besides, the low sample size at year 5 might cause some unusual observations to have an important influence on these effects, although no extreme observations were detected in %EBMIL. The predominance of female patients included within this study should be considered when generalizing these results across the general patient population. Furthermore, patients with major depressive disorder or other uncompensated psychiatric illness are not eligible for metabolic and bariatric surgery in Switzerland and therefore were not included in this analysis. This may have resulted in restricted range effects of the impact of psychiatric symptoms on weight loss outcomes.

## Conclusion

While follow-up after metabolic and bariatric surgery often focuses on the short-term outcomes, our findings underline the importance of careful long-term medical follow-up, especially of patients who suffer from anxiety and probably require closer medical surveillance. Our study also highlights the fact that weight loss and weight regain after bariatric surgery are difficult to predict based on the preoperative evaluation. There could be other preoperative or postoperative factors that should be taken into account so as to better predict the outcome or intervene in cases of high-risk of weight regain post-surgery. Further research is warranted to identify these factors with a prospect to improve the long-term medical care of bariatric patients.

## Data Availability

The data that support the findings of this study are avaiable on request from the corresponding author.
